# A Case of Hypernatremia in a Newly Diagnosed Patient With Acute Myeloid Leukemia: Lessons for Nephrologists

**DOI:** 10.7759/cureus.59186

**Published:** 2024-04-28

**Authors:** Valerio Rasi, Forest Riekhof, Maya Mahmoud, Shannon Ejiofor, Krista L Lentine

**Affiliations:** 1 Internal Medicine - Nephrology, Saint Louis University School of Medicine, Saint Louis, USA; 2 Internal Medicine, Baylor College of Medicine, Houston, USA

**Keywords:** arginine-vasopressin deficiency, hypernatremia, desmopressin, copeptin, acute myeloid leukemia (aml)

## Abstract

Arginine vasopressin deficiency (AVP-D), formerly known as central diabetes insipidus, is a disease characterized by polyuria, polydipsia, and hypernatremia. The concomitant diagnosis of acute myeloid leukemia (AML) is an underappreciated event that requires prompt recognition and treatment by practicing nephrologists and hematologists. This report highlights this importance by describing the case of a 39-year-old patient newly diagnosed with AML who developed severe hypernatremia. The role of diagnostic testing through desmopressin (DDAVP) challenge and copeptin testing to confirm the diagnosis of AVP-D in this context and the use of DDVAP for treatment are discussed. Practicing nephrologists and primary care providers taking care of patients with similar symptoms will benefit from understanding the pathophysiology of AVP-D, its relationship with AML, and the prognosis in this patient cohort.

## Introduction

Arginine vasopressin deficiency (AVP-D, formerly known as central diabetes insipidus, is a rare disorder that affects approximately one in 25,000 people in the general population [1,2]. The condition results from posterior pituitary dysfunction, resulting in a deficiency in the expression of arginine vasopressin (AVP) or antidiuretic hormone (ADH). The clinical presentation includes polyuria, nocturia, hypernatremia, and polydipsia. When hypernatremia is severe, patients can develop neurological disturbances, including encephalopathy. The cognate disease that AVP-D needs to be distinguished from is AVP resistance (AVP-R), formerly known as peripheral or nephrogenic central diabetes insipidus. Most cases of AVP-D are idiopathic, meaning the deficiency in AVP cannot be attributed to an infiltrative tumor of the posterior pituitary or a surgical etiology [3]. While tumor infiltration is usually reserved for metastatic disease, an association between AVP-D and acute myeloid leukemia (AML) has been identified [4]. Despite prior publications, many clinicians may be unfamiliar with the association due to uncommon clinical encounters. Herein, we describe the case of a 39-year-old patient with months of polydipsia admitted for induction therapy of AML in whom we made a new diagnosis of AVP-D. We review the approach to diagnosis and discuss implications important for practicing nephrologists and clinical teams.

## Case presentation

A 39-year-old Caucasian woman presented to the emergency department with splenomegaly and abdominal pain. A complete blood count demonstrated 20% blast cells, raising concern for acute leukemia. During admission, a bone marrow biopsy confirmed diagnosis of adverse risk AML, with karyotype: 45,XX,inv(3)(q21q26.2),-7; fluorescent in situ hybridization: monosomy 7, RPN1::MECOM fusion; and gene sequencing: SF3B1 mutated, KRAS mutated. The patient was started on induction chemotherapy 7+3 with cytarabine and idarubicin in the hospital. Of note, the patient presented with serum labs within a normal range and was not on any medications.

**Figure 1:**
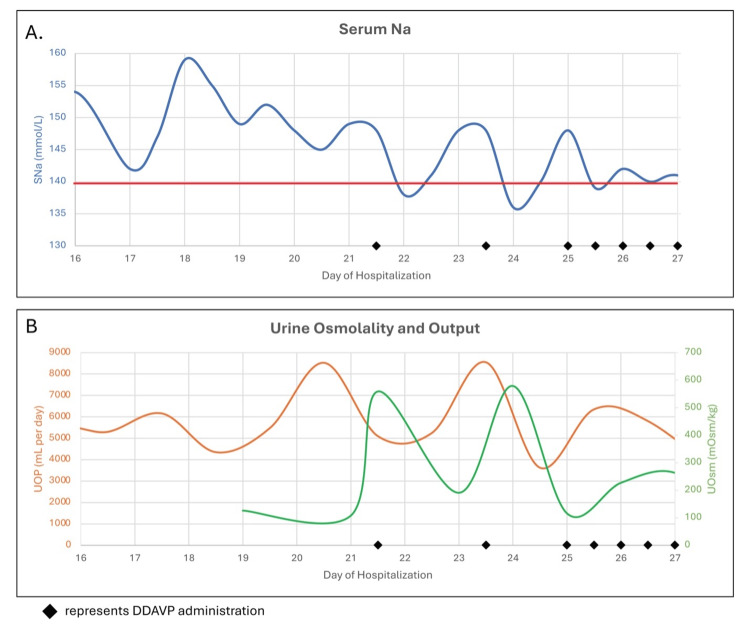
Evolution of hypernatremia and hyperosmolarity before and after DDAVP therapy A. In blue, serum sodium levels (SNA) expressed in mmol/L. In red, reference line showing 140 mmol/L. B. In orange, urine output (UOP) expressed in mL per day. In green, urine osmolality (UOsm) expressed in mOsm/kg. In black diamond, administration of desmopressin (DDAVP) during hospitalization.

During the hospitalization, the patient developed hypernatremia with serum sodium (SNa) reaching a peak level of 159 mEq/L at day 16 of hospitalization (D16), at which time nephrology service was consulted (Figure 1A). History taking during consultation elicited that the patient was experiencing polydipsia (drinking gallons of spring water without quenching her thirst) and polyuria for weeks prior to hospital admission, but had not sought evaluation for these symptoms, suggesting that hypernatremia may have been exacerbated by reduced access to water in the hospital setting. No neurological deficits were identified. Urine output (UOP) reached a maximum of 8.55 L/day with intake that was proportional to the output. Initial management included a 5% dextrose infusion at 150-175 mL/hour.

**Figure 2:**
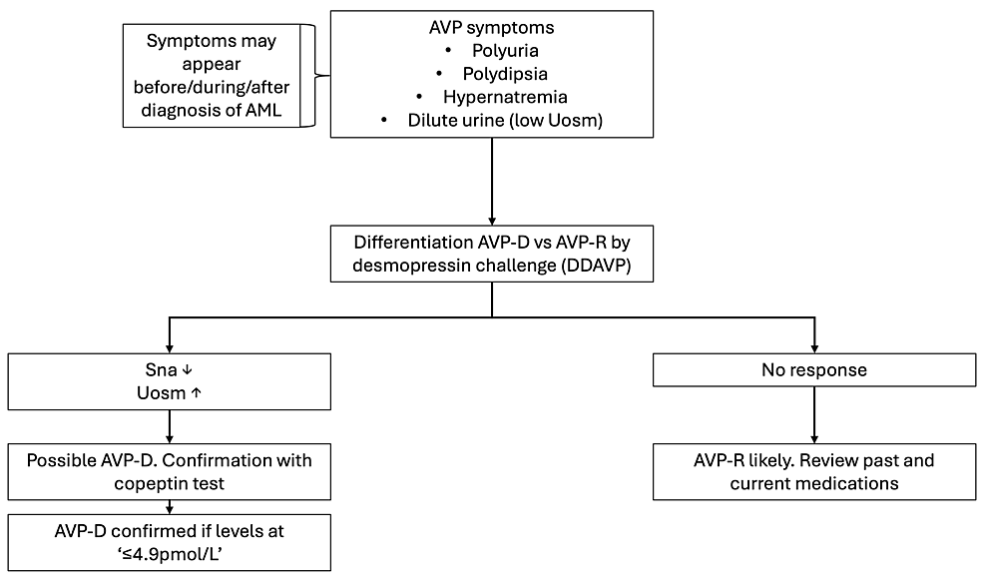
Clinical decision-making algorithm for the evaluation and confirmation of arginine vasopressin deficiency (AVP-D) in acute myeloid leukemia (AML) patients Arginine vasopressin resistance (AVP-R), desmopressin (DDAVP), serum sodium (SNa), urine osmolarity (UOsm)

**Figure 3:**
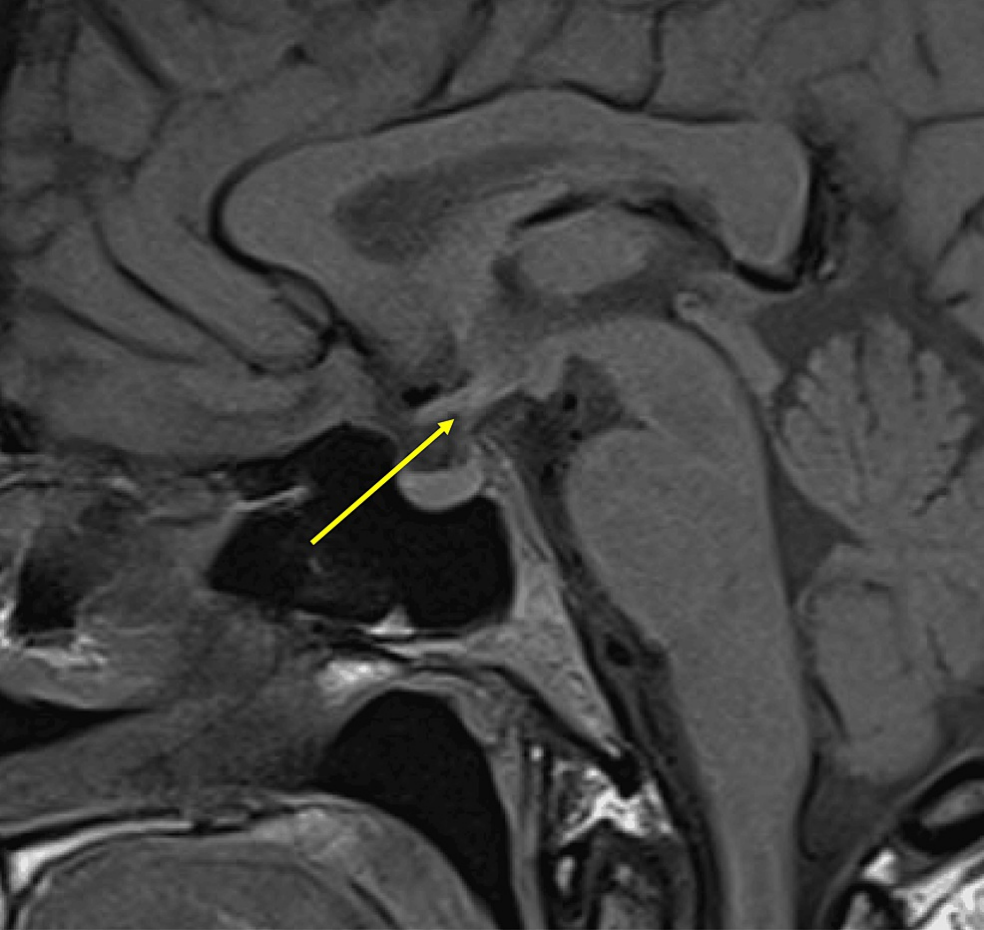
Magnetic resonance imaging section of the brain, sagittal T1 view, showing the absence of a posterior pituitary bright spot, with no evidence of posterior pituitary ectopia The yellow arrow indicates the location of the posterior pituitary, where the bright spot is absent.

Nephrology service evaluation included measurement of urine osmolality (UOsm), which was 126 mOsm/kg, on day 19. Given the findings of polyuria, hypernatremia, and inappropriately dilute urine, a diagnosis of AVP-D was contemplated (Figure 2). A desmopressin (DDAVP) challenge with 2 mcg intravenous was positive as the UOsm increased from 108 to 541 mOsm/kg and SNa decreased from 150 to 143 mEq/L (Figure 1B). A copeptin level was ordered to confirm deficiency in endogenous ADH production. Magnetic resonance imaging (MRI) of the brain with and without contrast focusing of the pituitary gland lacked the normal bright spot found in the posterior pituitary, which has been previously described in AVP-D (Figure 3).

Following the presumed diagnosis, the patient received intranasal administration of DDAVP on day 23 in consultation with the inpatient pharmacist. The patient again responded to treatment with a decline in SNa from 148 to 136 mEq/L and a decrease in UOP from 8.5 L to 3.6 L over the next 24 hours. Given the cost associated with intranasal administration, lower flexibility in dosing, and in discussion with the pharmacist, DDAVP dosing was changed to oral administration for long-term management. On day 25, the patient was transitioned to oral DDAVP 50 mcg twice daily, leading to serum sodium stabilization and polyuria improvement. SNa did not increase over 145 mEq/L, and the patient reported reduced thirst for the first time in months. The patient was able to continue appropriate hematology/oncology therapy. The patient consented to the description of her clinical information as a case report.

## Discussion

Hypernatremia in patients with prior/concurrent/late discovery of AML diagnosis has been previously reported [4,5]. The mechanism behind this association has not been identified, although there are reports highlighting a potential link with chromosome 3 inversion and MDS1 and EVI1 complex locus (MECOM) overexpression in the posterior pituitary, leading to the deficiency in the expression of ADH [5]. The goal of this case report is to increase recognition among nephrologists to facilitate prompt diagnostic testing and appropriate management. As shown in Figure 2, after recognition of symptoms consistent with AVP, it is important to understand the etiology of these symptoms and whether this is due to AVP-D vs. AVP-R.

AVP-D is caused by the lack of synthesis or secretion of AVP [6]. Copeptin is a cleavage product made during the synthesis of AVP, making it a biological marker for endogenous AVP synthesis [7]. The gold-standard test for this disease remains the DDAVP challenge test [2], as evidenced in our case, followed by copeptin levels. AVP-D must be differentiated from AVP-R. The established method for this differentiation relies on the response to DDAVP. In AVP-R, the DDAVP challenge does not increase UOsm and ameliorate hypernatremia due to resistance of DDAVP within the kidneys [2]. Lithium toxicity is one of the most common medications associated with AVP-R, and removal of this medicine from patients can improve resistance. It is important to address the endogenous deficiency of AVP in AVP-D with the use of DDAVP, instead of vasopressors, as DDAVP does not affect vasculature directly, but rather acts on the kidney to promote urinary concentration and water retention.

After the DDAVP challenge, if the patient responds to the treatment, then the copeptin test further confirms the diagnosis of AVP-D (Figure 2). If copeptin levels are ≤4.9 pmol/L, the diagnosis of AVP-D is confirmed, and treatment can be continued. We recommend oral DDAVP as treatment is easier to titrate. Starting with a dose of 50 mcg twice daily is reasonable. Doses up to 800 mcg per day appear to be tolerated but were not necessary in our patient [8]. Nasal spray can be used in treatment-refractory cases, but it is important to mention that each spray is approximately equivalent to 10 mcg, and precise administration is more difficult than oral administration [9].

Ladigan et al. [4] reviewed 51 reported cases of AVP-D in AML. In 41 of these cases, cytogenetic analysis was reported. The diagnosis of AVP-D in AML was highly coincident with a monosomy 7 karyotype, 75.6 %, or inv(3)(q21q26), 46.3 %; of the cases with monosomy 7, 54.8 % also harbored chromosome 3 aberrations. It remains unknown if AVP-D is an independent and adverse prognostic factor in AML [10]. Nevertheless, with the young age, a median of 48 years in this series, allogeneic hematopoietic cell transplant (alloHCT) should be pursued in this patient population. Out of nine cases of AML with AVP-D who achieved complete AML remission (independent of alloHCT or chemotherapy), seven patients (78%) did not require DDAVP therapy any longer [4]. These data suggest the association between AML and AVP-D development and its reversal following treatment.

## Conclusions

In conclusion, it is important for nephrologists to recognize that hypernatremia in AML patients can be due to a new diagnosis of AVP-D, which can prompt diagnosis and treatment. Future studies are needed to discern if prompt recognition of AVP-D increases the overall survival of AML patients and improves treatment outcomes.
